# Identification of Glypican-3 as a potential metastasis suppressor gene in gastric cancer

**DOI:** 10.18632/oncotarget.9763

**Published:** 2016-06-01

**Authors:** Shiwei Han, Xuemei Ma, Yanxia Zhao, Hongying Zhao, Ana Batista, Sheng Zhou, Xiaona Zhou, Yao Yang, Tingting Wang, Jingtao Bi, Zheng Xia, Zhigang Bai, Igor Garkavtsev, Zhongtao Zhang

**Affiliations:** ^1^ Department of General Surgery, Beijing Friendship Hospital, Capital Medical University, Beijing, China; ^2^ Department of Radiation Oncology, Massachusetts General Hospital, Boston, MA, USA; ^3^ Beijing Key Laboratory of Cancer Invasion and Metastasis Research & National Clinical Research Center for Digestive Diseases, Beijing, China; ^4^ Cancer Center, Union Hospital, Tongji Medical College, Huazhong University of Science and Technology, Wuhan, China; ^5^ Department of Pathology, Beijing Chaoyang Hospital, Capital Medical University, Beijing, China; ^6^ Institute of Pathology, Union Hospital, Tongji Medical College, Huazhong University of Science and Technology, Wuhan, China; ^7^ Department of General Surgery, Beijing Jishuitan Hospital, The Fourth Medical College of Peking University, Beijing, China; ^8^ Department of Surgery, Xiangya Hospital, Central South University, Changsha, China

**Keywords:** gastric cancer, Glypican-3, metastasis, FoxM1, Erk1/2

## Abstract

Gastric cancer is a prevalent tumor that is usually detected at an advanced metastatic stage. Currently, standard therapies are mostly ineffective. Here, we report that Glypican-3 (GPC3) is absent in invasive tumors and metastatic lymph nodes, in particular in aggressive and highly disseminated signet ring cell carcinomas. We demonstrate that loss of GPC3 correlates with poor overall survival in patients. Moreover, we show that absence of GPC3 causes up-regulation of MAPK/FoxM1 signaling and that blockade of this pathway alters cellular invasion. An inverse correlation between GPC3 and FoxM1 is also shown in patient samples. These data identify GPC3 as a potential metastasis suppressor gene and suggest its value as a prognostic marker in gastric cancer. Development of therapies targeting signaling downstream of GPC3 are warranted.

## INTRODUCTION

Gastric cancer is the third most prevalent solid tumor in adults [1]. Most patients are diagnosed with advanced non-operable or metastatic diseases and, therefore, have very poor outcomes [2]. Consequently, only about 3% of patients with metastatic cancer achieve 5-year survival [2, 3]. Gene expression studies in gastric cancer have shown alterations in the transcription levels [4] of Glypican-3 (GPC3), a member of the glypican family of heparin sulfate proteoglycans [5]. Specifically, GPC3 mRNA levels are significantly lower in tumors as compared to normal gastric tissues [4]. In addition, the protein levels of GPC3 in patients with gastric malignancies are also decreased and often undetectable by immunostaining compared to hepatocellular carcinoma [6], where in hepatocellular carcinoma GPC3 behave as oncogene and very often has high expression level compared to normal livers [7]. In normal physiology, GPC3 localizes to the cell surface, where it participates in the regulation of cell growth and cell division [8, 9]. Loss-of-function mutations in GPC3 cause the rare X-linked Simpson-Golabi-Behmel syndrome (SGBS) [10]. Importantly, patients with SGBS are at increased risk of developing cancer, predominantly in the abdominal region [11, 12]. Similarly, mice deficient in GPC3 also have developmental overgrowth features [8]. In breast cancer, changes of GPC3 expression regulate invasion and metastasis [9]. However the role of GPC3 in gastric cancer is not elucidated and fully understood. Here, we evaluate the function of GPC3 in gastric tumors, in particular its ability to regulate mechanisms of tumor cell invasion and metastatic spread.

## RESULTS

### Low expression of GPC3 correlates with metastasis and low prognosis in gastric cancer patients

**Figure 1:**
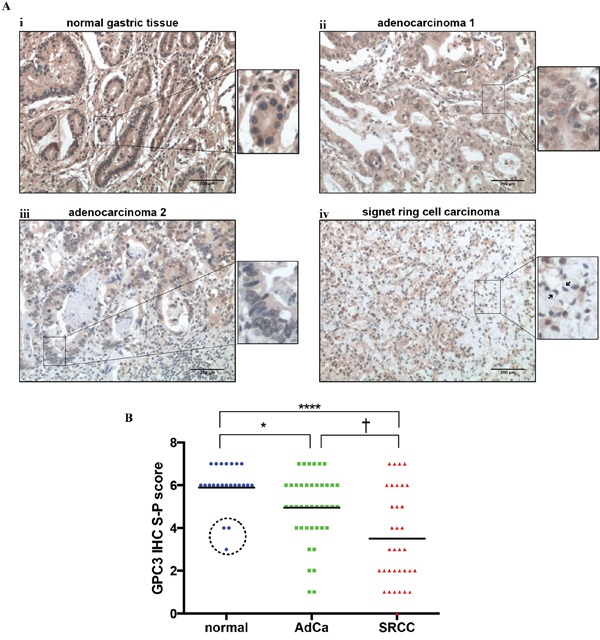
GPC3 protein expression is lower in gastric cancer than in normal gastric tissue Expression of GPC3 protein was detected in 75 primary gastric tumors, 11 paired adjacent pre-cancerous tissues and 12 normal gastric tissues by IHC. **A.** Representative images illustrate ***i***: strong GPC3 staining (brown) in normal tissue; ***ii***: strong GPC3 staining in adenocarcinoma tissue; ***iii***: weak GPC3 staining in adenocarcinoma tissue; and ***iv***: weak and negative GPC3 staining in signet ring cell carcinoma tissue. Highly magnification images show cellular staining of GPC3 for each panels (arrows indicate signet ring cells). **B.** Quantification of GPC3 expression by IHC S-P score. (AdCa: adenocarcinoma; SRCC: signet ring cell carcinoma; dashed circle: adjacent precancerous tissues of signet ring cell carcinoma; *, P=0.0384, †, P=0.0315, ****, P<0.0001, Dunn's).

To examine the expression levels of GPC3 in gastric cancers, we stained 75 gastric tumors (41 adenocarcinomas and 34 signet ring cell carcinomas), 11 adjacent pre-cancerous tissues, and 12 normal gastric tissues with antibodies raised against GPC3 protein. None of the patient had received neoadjuvant treatment (chemo- and radio-therapy) prior surgery. GPC3 protein was highly expressed in normal tissues (Figure 1A-i) and, in all pre-cancerous tissues except for tissues adjacent to signet ring cell carcinoma (Figure 1B dashed circle). In normal tissues, GPC3 was expressed in almost every cell type (mucous, gastric glands, gastric smooth muscle cells). Although adenocarcinoma samples stained positive, the level of GPC3 was low as compared to controls in 59% of tumors (24/41) (Figure 1A-ii- high expression, Figure 1A -iii- low expression, Figure 1B). In contrast, GPC3 expression in signet ring cell carcinomas is much lower than both normal tissues and adenocarcinomas, shown as 74% of signet ring cell carcinomas (25/34) stained weak or non-detectable levels of GPC3 (Figure 1A-iv). The IHC S-P score was used for quantification of GPC3 expression in gastric tissues (Figure 1B). Together, the preceding observations indicate that the level of GPC3 protein was low in gastric cancer patients' tissues compared to controls.

**Figure 2:**
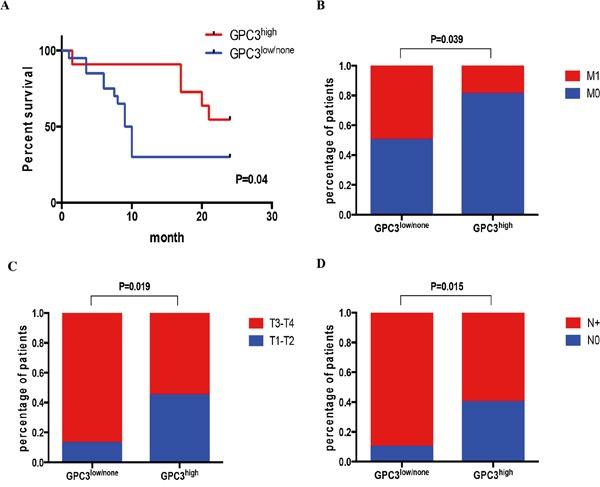
Low GPC3 expression correlates with metastasis and poor survival Patients were classified as GPC3^high^ or GPC3^low/no^ by IHC S-P score. **A.** Kaplan-Meier curve shows that high GPC3 expression correlates with better survival (N=31, P=0.04, Gehan-Breslow-Wilcoxon test). Fisher's exact test showed that patients with GPC3^low/no^ have **B.** more metastasis (N=51, P=0.0389); **C.** have advanced T3-T4 stage metastatic invasion (N=51, P=0.0194); and **D.** have more lymph node with metastasis (N=51, P=0.0154). (M0: no distant metastasis; M1: has distant metastasis; T1: tumor invades lamina propria, muscularis mucosae, or submucosa; T2: tumor invades muscularis propria; T3: tumor penetrates subserosal connective tissue without invasion of visceral peritoneum or adjacent structures; T4: tumor invades serosa or adjacent structures; N0: no regional lymph node metastasis; N+: have regional lymph node metastasis)

To analyze the link between expression level of GPC3 in gastric cancer and patient overall survival, we compared GPC3 levels in 31 patients (Beijing cohort). This cohort included patients with adenocarcinomas (n=25) and signet ring cell carcinomas (n=6). We found that low expression of GPC3 in tumors significantly correlated with decreased overall survival of patients (Figure 2A, P=0.04). The median survival of patients with low GPC3 expression was 9.5 months, while patients with high GPC3 expression survived more than 24 months. GPC3 expression did not correlate with tumor size or differentiation (Table 1), and this result further confirmed with *in vitro* and *in vivo* experiments (supplementary Figure 1). However, there was significant association between lower levels of GPC3 and higher number of distant metastases (Figure 2B, P=0.039), depth of invasion (Figure 2C, P=0.019), and tumor spread to the lymph nodes (Figure 2D, P=0.015) (n=51, 31 cases of Beijing cohort, 20 cases of Wuhan cohort). These data indicate that GPC3 expression is reduced in tumors compared to normal gastric tissues, and patients with primary tumors with low GPC3 have more metastasis and worse prognosis.

**Table 1:** Relationship of GPC3 Expression and Pathologic Features of Gastric Cancer

Variables	N	GPC3 expression		P
		Low/Non	High	
Age (y)				NS
<55	23	14	9	
≥55	28	17	11	
Sex				NS
Male	33	18	15	
Female	18	13	5	
Tumor size (cm)		4.62±1.45	5.31±1.87	NS
Differentiation				NS
Moderate-Well	14	7	7	
Poorly-Non	37	24	13	
T stage				0.0194
T_1_-T_2_	13	4	9	
T_3_-T_4_	38	27	11	
N stage				0.0154
N_0_	11	3	8	
N_+_	40	28	12	
M stage				0.0389
M_0_	32	15	17	
M_1_	19	15	4	

NS, not significant; P value was calculated by Fisher's exact test or Student's t-test

### GPC3 expression is low in lymph node metastasis

**Figure 3:**
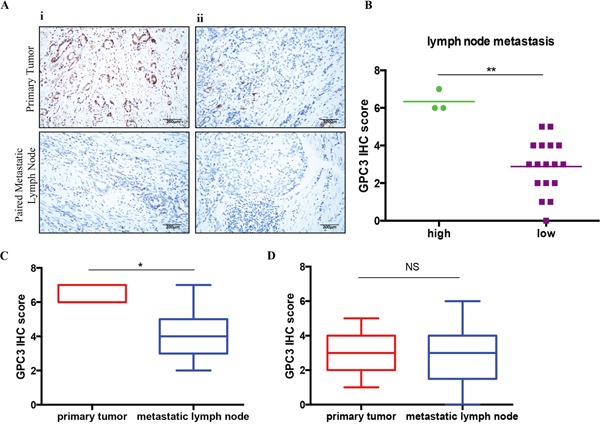
Lost of GPC3 in metastatic lymph nodes GPC expression was detected in 20 primary gastric tumors and paired metastatic lymph nodes by IHC. **A.** Representative images illustrate GPC3 staining for ***i***: GPC3^high^ primary tumor and ***ii***: GPC3^low^ primary tumor (upper) with paired metastatic lymph nodes (lower). **B.** Quantification of GPC3 expression in metastatic lymph nodes by IHC S-P score. (**, P=0.0018, Mann-Whitney t-test). **C.** GPC3 high expressed primary tumors have significant low GPC3 expression in the paired metastatic lymph nodes (*, P=0.0469, Wilcoxon paired t-test). **D.** GPC3 low expressed primary tumors have low GPC3 expression in the paired lymph nodes (NS: not significant, Wilcoxon paired t-test).

To test if metastatic lesions have low GPC3 levels independently of the expression in the original lesions, we stained GPC3 in paired primary tumors and metastatic lymph nodes (n=20). We confirmed metastasis in lymph nodes by H&E staining and evaluated by pathologists (H&E staining data not show). We observed that although a few metastatic lymph nodes had high GPC3 expression, 85% (17/20) of metastatic nodes had low level staining for GPC3 (Figure 3A, 3B). GPC3^high^ primary tumors lost GPC3 in the metastatic setting (Figure 3C, P=0.04). Conversely, GPC3^low^ primary tumors maintained low expression of GPC3 in metastasis, shown as no statistic difference in GPC3 expression between GPC3^low^ primary tumors and the paired metastatic lymph nodes (Figure 3D, P=0.82). Collectively, these data indicate that metastatic cells express low level of GPC3 despite the GPC3 status in the primary lesions.

### Loss of GPC3 increases the invasion of gastric tumor cells through activation of MAPK/FoxM1 signaling

**Figure 4:**
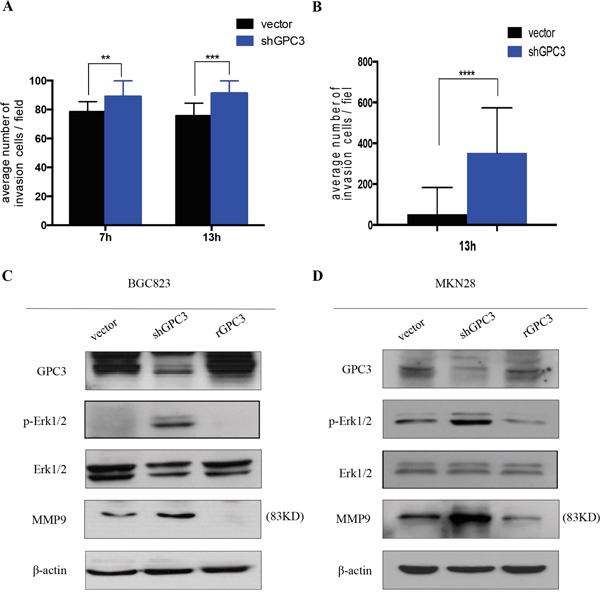
Loss of GPC3 increases the invasion of gastric tumor cells Invasion assay in **A.** BGC823 cells and **B.** MKN28 cells transfected with shGPC3 (blue bar) and control vector (black bar). At different time points (7 hours and/or 13 hours), shGPC3 cells exhibit increased invasion ability (**, p=0.0036; ***, p=0.0006; ****, p<0.0001. unpaired t-test). Immunoblot reveals the signal changing after GPC3 knockdown by shGPC3 and GPC3 overexpression by transfecting with human recombinant GPC3 in **C.** BGC823 cells and **D.** MKN28 cells.

To evaluate if the loss of GPC3 affects the metastatic behavior of tumor cells, we decreased the expression of GPC3 in BGC823 and MKN28 gastric tumor cell lines with shRNA (sh-GPC3) and conducted matrigel invasion assays. Downregulation of GPC3 expression significantly increased the invasive capacity of tumor cells compared to controls (Figure 4A, BGC823, 7 hrs p=0.0036, 13 hrs p=0.0006; Figure 4B, MKN28, 13 hrs, p<0.0001). These results suggest that GPC3 is involved in the regulation of invasion programs in gastric tumors.

**Figure 5:**
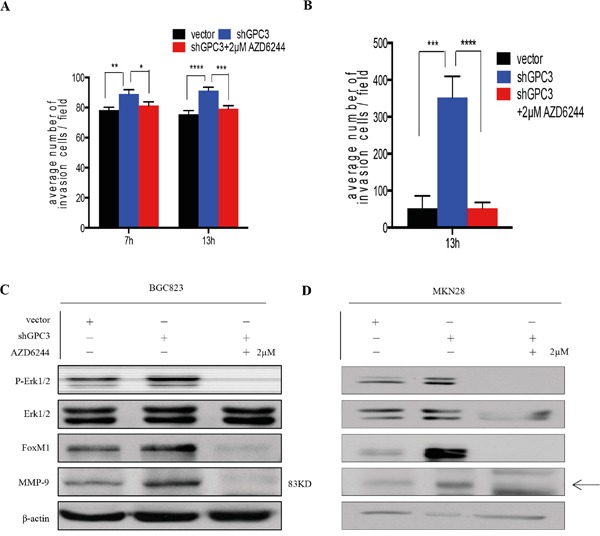
Erk inhibition abrogates the invasion ability of gastric cancer cells with GPC3 knockdown **A.** 2μM AZD6244 treatment abrogated shGPC3-BGC823 cells invasion ability (red bar) in different time points (7 hours, *, P<0.05, **, p<0.01; 13 hours, ***, P<0.001, ****, P<0.0001; one-way ANOVA); **B.** 2μM AZD6244 treatment abrogated shGPC3-MKN28 cells invasion ability (red bar) (***, p<0.001; ****, p<0.0001, one-way ANOVA); inhibition in Erk1/2 signaling leads to downregulation FoxM1 and MMP9 in both **C.** BGC823 shGPC3 cells and **D.** MKN28 GPC3 cells.

To understand how the loss-of-GPC3 increases the capacity of tumor cells to invade, we analyzed the activation status of MAPK signaling, a pathway involved in the regulation of invasion and metastasis [13]. We found that repression of GPC3 in BGC823 and MKN28 cells increases phosphorylation of Erk1/2 (Thr202/Tyr204). In contrast, overexpression of GPC3 in both cell lines causes a reduction of phosphorylated Erk1/2 (Figure 4C, Figure 4D). In addition, we found that MMP9 expression is increased in cells that have repressed GPC3, and overexpression of GPC3 reduces the expression of MMP9 (Figure 4C, Figure 4D). To determine whether loss-of-GPC3 mediated invasion was dependent on the phosphorylation of Erk1/2 signaling, we treated shGPC3 BGC823 and MKN28 cells with MEK inhibitor (AZD6244). We observed that inhibition of MEK decreased the number of invasive tumor cells through the matrigel (Figure 5A, BGC823, 7 hrs p<0.05, 13 hrs p<0.001; Figure 5B, MKN28, 13 hrs, p<0.0001).

MMP9 is a known target of FoxM1, a key transcription factor that regulates tumor invasiveness and metastasis [14, 15]. Moreover, evidences show FoxM1 as a downstream target of the Ras/ERK/MAPK signaling pathway [16–18]. Thus, we checked levels of FoxM1 in cells with different expression levels of GPC3 and cells treated with AZD6244. We found that silencing of GPC3 induces expression of FoxM1 in gastric tumor cells, and that is abrogated by MEK inhibition (Figure 5C, Figure 5D). Together, these findings suggest that loss of GPC3 activates the Erk1/2-FoxM1-MMP9 signaling axis, thereby promoting cancer cell dissemination.

### GPC3 expression negatively correlates with FoxM1 expression in primary gastric tumors

**Figure 6:**
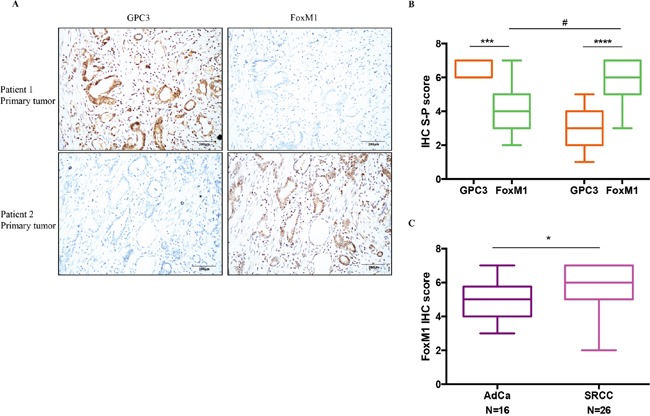
The negative correlation between GPC3 expression and FoxM1 expression in patient tumors Expression of GPC3 and FoxM1 were detected in 42 primary gastric tumors by IHC. **A.** IHC staining for GPC3 (left) and FoxM1 (right) in the same tumor region of patient 1 (upper) and patient 2 (lower). **B.** Quantification of GPC3 expression and FoxM1 expression by IHC S-P score. (***, P=0.0005, Wilcoxon paired T-test; ****, P<0.0001, Wilcoxon paired t-test; #, P=0.0001, Mann-Whitney t-test). **C.** FoxM1 IHC S-P score for adenocarcinoma (AdCa) and signet ring cell carcinoma (SRCC). (*, P=0.015, Mann-Whitney t-test).

To verify the relevance of GPC3-mediated regulation of FoxM1 in gastric tumors, we performed IHC staining for GPC3 and FoxM1 in 42 primary samples (Wuhan cohort). We found that GPC3 expression inversely correlates with the expression of FoxM1 (Figure 6A). IHC score shows significant negative correlation between expression of GPC3 and FoxM1 (Figure 6B, ***, p=0.0005; ****, P<0.0001). Levels of FoxM1 in GPC3^low^ tumors are significantly higher than in GPC3^high^ tumors, showing an absolute, but not relative increase of FoxM1 (Figure 6B, #, P=0.0001). Signet ring cell carcinoma, the subtype with the lower overall expression of GPC3 than adenocarcinoma (Figure 1B), shows significantly higher levels of FoxM1 compared to adenocarcinoma (Figure 6C, p=0.015). In conclusion, the loss of GPC3 causes an up-regulation of FoxM1 in gastric cancers.

## DISCUSSION

Earlier reports examining the transcriptional profile of GPC3 showed marked gene down-regulation in gastric tumors, suggesting a potential tumor suppressor role for GPC3 [4]. However, the precise role of GPC3 in gastric tumor development and progression remains unclear due to the paucity of both pre-clinical and clinical correlative studies. Later studies evaluating GPC3 protein levels in a small cohort of gastric adenocarcinomas (n=8) and neuroendocrine carcinomas (n=8) suggested that low expression of GPC3 did not correlate with tumor size or tumor differentiation [6], therefore implying GPC3 in gastric oncogenesis but not in the regulation of primary tumor growth. In this work, we identify GPC3 as a potential metastasis suppressor gene that controls cellular mechanisms of invasion and metastasis in gastric tumors. Specifically, we have demonstrated that 1) GPC3 has a prognostic value and low expression levels correlate with poor clinical outcome; 2) loss of GPC3 increases the invasive capacity of tumor cells; 3) GPC3 expression is lost in metastatic lesions in the lymph nodes regardless of GPC3 expression in primary tumors; 4) GPC3 signals downstream to MAPK/FoxM1 to regulate the cellular invasion program and 5) GPC3 and FoxM1 inversely correlate in primary gastric tumors.

Our retrospective analysis of an annotated cohort of gastric tumors establishes an important prognostic value for GPC3. In accordance with previous studies [6], we did not find a significant correlation between GPC3 levels and tumor size or tumor differentiation. Instead, we uncovered a strong correlation between GPC3 and the incidence of local and distant metastasis and presence of lymph nodes metastasis. Therefore, GPC3 does not seem to be involved in the development of gastric tumors, but it likely impacts disease progression by regulating metastatic spread. Of note, the study cohort consisted of untreated tumors, precluding any secondary confounding effects of radiation and chemotherapy in the evolution of tumors. We speculate that GPC3 will be less expressed in distant metastasis than in primary lesions. Given the difficulty in obtaining biopsies from distant metastasis (primarily liver and lung metastasis), we have analyzed surgical resected metastatic lymph nodes --- the intermediate sites of colonization between the primary and secondary organs. It will be important to confirm these results not only in larger patient cohorts, but also specially in distant metastatic lesions. This will require the creation of metastatic biopsies/autopsy banks. Available mouse models (orthotopic xenografts) do not recapitulate human tumors since they rarely metastasize [19, 20]. Next generation genetic engineered mouse models (GEMM), with conditional switch-off of GPC3 in the gastric tissue, will be very valuable. Currently, such models do not exist.

The most striking finding of our study is the almost complete absence of GPC3 in signet ring cell carcinomas, tumors that histologically more diffuse than other gastric cancer types [21, 22]. In these tumors, even pre-cancerous tissues express lower level of GPC3. This suggests that analyzing GPC3 levels may aid in the early detection of cancer, in particular signet ring cell subtype. Equally importantly, levels of GPC3 can potentially be used as a diagnostic marker to define more invasive and aggressive gastric cancers and, therefore, be valuable to assign patients to more tailored treatment regiments.

The mechanisms that lead to loss of GPC3 are not elucidated. Studies in ovarian cancer cell lines uncover that the silencing of GPC3 is regulated epigenetically rather than through genetic mutations [23]. We observed that there is loss of GPC3 in tumors compared to normal gastric tissues. Whether this occurs through epigenetic or genetic mechanisms needs to be further investigated.

Molecular studies revealed the activation of MAPK kinases and FoxM1 after loss of GPC3 in tumor cells. FoxM1 is a key transcription factor in cancer implicated in the regulation of angiogenesis, migration, invasion and metastasis [24]. Several studies suggest that mono- or combination therapies targeting FoxM1 can have potent anti-tumor effects [25, 26]. However, challenges in drug delivery, toxicity and bioavailability are still hampering the development of efficacious FoxM1 inhibitors [15, 27]. Conversely, several MEK inhibitors are currently in clinical trials and the toxicity profile is moderate [28]. At the moment, there are no trials utilizing MEK inhibitors in gastric cancer. According to the results presented here, we anticipate that MEK inhibitors may be of value in gastric cancer types where GPC3 is absent. Further studies in larger patient cohorts to evaluate the activation status of MAPK in gastric tumors are warranted. Although we identified a link between GPC3 and MAPK/FoxM1, the exact molecular mechanism that leads to activation of Erk1/2 is unknown. In other tumor types, such as hepatocellular carcinoma, where GPC3 functions as an oncogene, GPC3-mediated oncogenesis involves the activation of Wnt or Insulin-like growth factor signaling [29, 30]. Development of targeted therapies for gastric tumors or repurposing available drugs will require a more complete understanding of the signaling pathways involved these cancers.

In summary, we have found that GPC3 involved in the metastatic process, and GPC3 repression correlates with poor prognosis for patients with gastric cancer. The role of GPC3 in the progression of invasive tumors supports the consideration of drugs that interfere with MAPK/FoxM1 pathways for a subset of gastric cancer patients.

## MATERIALS AND METHODS

### Primary samples and cell lines

Samples were collected under a study protocol approved by the Institutional Review Board of Beijing Friendship Hospital, Capital Medical University, Beijing, China (Beijing cohort, 31 patients) and under a study protocol approved by the Institutional Review Board of Tongji Hospital, Tongji Medical College, Huazhong University of Science and Technology, Wuhan, China (Wuhan cohort, 44 patients). Tumor tissues and lymph nodes were collected at time of surgery from neoadjuvant naive patients (without prior chemotherapy and radiation). Patients underwent D2 distal, subtotal or total gastrectomy, according to the extent of disease. Tumor stage (TNM) was determined post-resection according to the American Joint Committee on Cancer classification for stomach carcinoma. Pre-cancerous paired tissues were also collected from 11 patients. Normal gastric tissues (n=12) were collected from patients undergoing routine gastroscopy. All tissue specimens were fixed with formalin, embedded in paraffin and tissue type of confirmed by standard H&E histology. Patients in the Beijing cohort were followed-up with every 4 months, for a total of 6 visits.

Human BGC823 cell line (undifferentiated gastric carcinoma) was obtained from the China Center for Type Culture Collection (Shanghai, China) and kept in DMEM medium supplemented with 10% fetal bovine serum (FBS). Human MKN28 cell line (moderately differentiated gastric carcinoma) was kindly provided by Dr. Susan Hagen (Beth Israel Deaconess Medical Center, Harvard Medical School, Boston) and maintained in RPMI medium with 10% FBS.

### Immunohistochemistry

Tissue sections (4μm) were air-dried, deparaffinized and rehydrated. Antigen retrieval was done with EDTA (pH 9.0) in a pressure cooker for 15 mins. Endogenous peroxidase activity was blocked for 20 min with H_2_O_2_ (3%). Slides were incubated at room temperature for 1h with primary antibody (anti-GPC3, Abgent, RB0503, 1:100; anti-FoxM1, Abcam, ab100806, 1:100) and 30 min with species-specific secondary antibody. The reaction was developed with DAB (Dako) and slides were counterstained with hematoxylin. Staining was evaluated blindly by four pathologists (HZ and XZ evaluated the Beijing cohort, and SZ and YZ evaluated the Wuhan cohort). A minimum of 10 fields/slide was examined with ×20 magnification. Slides were scored according to signal intensity and number of positive cells: score 0 (−); score 1-2 (+); score 3-5 (++); score 6-7 (+++). Tissues were graded as having low expression (score 0-5) or high expression (score 6-7).

### Immunoblot

Total cell lysates were prepared in RIPA buffer with added protease and phosphatase inhibitors (Roche). Protein lysates (30 μg) were resolved in reducing SDS/PAGE. Antibodies were used according to manufacturer's recommendation: Cell Signaling (Erk1/2, 9102; pErk1/2, D13.14E, 4370; FoxM1, D12D5, 5436); Millipore (GPC3, 9C2, MABC667; MMP9, 56-24A, MAB3309); Abgent (GPC3, RB0503, AP6337a); Sigma (β-Actin, AC-15, 122M4782). For MAPK inhibition, cells were treated with 2μM AZD6244 for 24hrs.

### Invasion assays

Cell migration was determined across 8μm size Transwell inserts coated with matrigel (100μl 1:1 dilution with serum-free medium). Cells (5×10^4^) were resuspended in serum-free medium and added to the upper chamber. Medium supplemented with 10% serum was added to the lower chamber. To block MAPK signaling, 2μM AZD6244 was added into the cell suspension. After incubation (7hrs, 13hrs), the invasive cells were fixed with 3.7% formaldehyde, stained with Gimsa, and counted. Three independent experiments of triplicates were performed. Five random images were taken per chamber and data were expressed as mean ± SEM.

### ShRNA, and overexpression studies

Short oligomers targeting human GPC3 (NM_00164617) were chemically synthesized (Invitrogen) and inserted into the pLVX-shRNA2 lentiviral vector containing GFP (Clontech) (sense strand sh1-5′-GATCCctcgagAAAAAAGAGCAAGACGTGACCTGAAAGtctcttgaa CTTTCAGGTCACGTCTTGCTC-3′; sh2-5′-GATCCctcgagAAAAAAGGCTCTGAATCTTGGA ATTGAtctcttgaaTCAATTCCAAGATTCAGAGCC-3′; sh3-5′-GATCCctcgagAAAAAAGCCGAA TGCTCACCAGAATGTtctcttgaaACATTCTGGTGAGCATTCGGC-3′; sh4-5′-GATCCctcgagAAA AAAGCGGTTACTGCAATGTGGTCAtctcttgaa TGACCACATTGCAGTAACCGC-3′). Lentiviral particles were produced in 293T cells and gastric cancer cell lines were infected according to manufacturer's protocol. Cells expressing shRNA constructs were selected as the 5% brightest GFP^+^ population.

To overexpress GPC3, the coding sequence of human transcript variant 1 was amplified from a cDNA library with Pyrobest DNA Polymerase (Takara, Japan) and cloned into the mammalian expression vector pEGFP-C2 (Clontech) between EcoRI and SalI sites.

### Statistical analysis

Clinical parameters in primary samples were compared by Fisher's Exact Test or Student's T Test, as appropriate. Survival curves were determined using the Kaplan-Meier method and differences between groups were estimated by the Gehan-Breslow-Wilcoxon test. Statistical significance between groups in normally distributed continuous variables was determined by using paired or unpaired T-test; one-way ANOVA or Dunnett's *post hoc* test. In non-Gaussian distributed variables, the statistical significance between groups was determined using Mann-Whitney test followed by Dunn's multiple comparisons *post hoc* test or Wilcoxon matched-pairs signed rank test. Statistical significance was achieved with p-values ≤ 0.05. Statistical analysis was done with Prism 6.0 Software.

## SUPPLEMENTARY FIGURE


