# Supplementary material for: Identification of Glypican-3 as a potential metastasis suppressor gene in gastric cancer
Source: Oncotarget. 2016 Jun 1;7(28):44406–16. doi: 10.18632/oncotarget.9763 (PMC5190106; doi:10.18632/oncotarget.9763)
Supplement: Supplementary file 1 [file oncotarget-07-44406-s001.pdf]

Identification of Glypican-3 as a potential metastasis suppressor gene in gastric cancer

SUPPLEMENTARY FIGURE

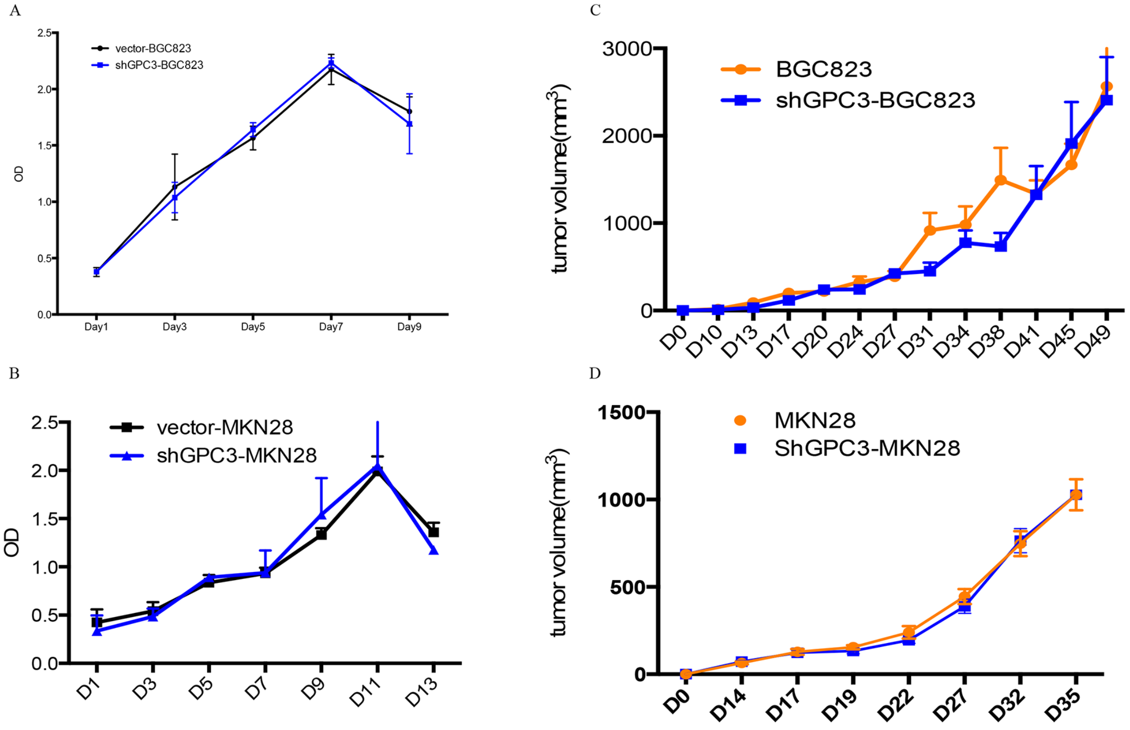

Supplementary Figure S1: GPC3 expression does not correlate with tumor growth. MTT assay showed that GPC3 knockdown did not affect on **A.** BGC823 cells or **B.** MKN28 cells growth. Subcutaneous tumor model showed that GPC3 knockdown did not affect on **C.** BGC823 or **D.** MKN28 tumor growth *in vivo* (n=6 for each group).
